# Clinician Perspectives of COVID-19-Related Cancer Drug Funding Measures in Ontario

**DOI:** 10.3390/curroncol28020103

**Published:** 2021-02-26

**Authors:** Rohini D. Naipaul, Rebecca E. Mercer, Kelvin K. W. Chan, Lyndee Yeung, Leta Forbes, Scott Gavura

**Affiliations:** 1 Provincial Drug Reimbursement Programs, Ontario Health (Cancer Care Ontario), Toronto, ON M5G 2L3, Canada; rohini.naipaul@ontariohealth.ca (R.D.N.); rebecca.mercer@ontariohealth.ca (R.E.M.); kelvin.chan@sunnybrook.ca (K.K.W.C.); lyndee.yeung@ontariohealth.ca (L.Y.); leta.forbes@ontariohealth.ca (L.F.); 2 Canadian Centre for Applied Research in Cancer Control, Toronto, ON M5G 2L3, Canada; 3 Odette Cancer Centre, Sunnybrook Health Sciences Centre, Toronto, ON M4N 3M5, Canada

**Keywords:** cancer drug reimbursement, clinical decision making, COVID-19, medical oncology, survey and questionnaire

## Abstract

The COVID-19 pandemic has a significant impact on cancer patients and the delivery of cancer care. To allow clinicians to adapt treatment plans for patients, Ontario Health (Cancer Care Ontario) issued a series of interim funding measures for the province’s New Drug Funding Program (NDFP), which covers the cost of most hospital-delivered cancer drugs. To assess the utility of the measures and the need for their continuation, we conducted an online survey of Ontario oncology clinicians. The survey was open 3–25 September 2020 and generated 105 responses. Between April and June 2020, 46% of respondents changed treatment plans for more than 25% of their cancer patients due to the pandemic. Clinicians report broad use of interim funding measures. The most frequently reported strategies used were treatment breaks for stable patients (62%), extending dosing intervals (59%), and deferring routine imaging (56%). Most clinicians anticipate continuing to use these interim funding measures in the coming months. The survey showed that adapting cancer drug funding policies has supported clinical care in Ontario during the pandemic.

## 1. Introduction

The implications of the 2019 novel coronavirus disease (COVID-19) pandemic are of significant concern to the cancer community. Evidence that has emerged to date suggests cancer patients may be at a higher risk of contracting SARS-CoV-2, may have an increased risk of developing severe COVID-19 illness, and may have a higher fatality rate compared to the general population [1,2,3,4,5,6,7]. Accordingly, prescribers are challenged with balancing the risks and benefits of treating cancer patients who are also impacted, both directly and indirectly, by COVID-19. As countries try to contain the spread of COVID-19 and manage active COVID-19 infections, cancer patients may face treatment interruptions or delays in accessing standard diagnostic procedures and treatment modalities, including curative surgeries, radiation, and systemic therapies [8,9,10,11,12].

In Ontario, the Ministry of Health has the overall responsibility for the funding and delivery of health care to residents. Cancer Care Ontario, now part of Ontario Health (OH(CCO)), is the provincial agency responsible for planning and funding most cancer services. Over 70 hospital sites deliver systemic treatment (e.g., parenteral chemotherapy, immunotherapy) in outpatient clinics. Hospitals are organized into 14 different regions with different levels of care offered within each region. In early March 2020, access to healthcare in Ontario was anticipated to be severely impacted by lockdown measures needed to contain the spread of COVID-19 and to treat COVID-19 patients in hospitals. As a result, OH(CCO) issued cancer-specific clinical guidance on the pandemic [13,14]. In parallel, OH(CCO) adapted its funding policies for systemic therapy for drugs funded through the New Drug Funding Program (NDFP), which is the primary funding program for injectable cancer therapies administered in outpatient hospital clinics. To qualify for public funding, patients must satisfy explicit clinical criteria. However, given the significant impact of COVID-19 on the delivery of cancer care, satisfying these criteria may not be optimal or even achievable.

Between 31 March and 31 July 2020, OH(CCO) issued a series of interim funding measures for NDFP-funded drugs to allow clinicians to modify treatment plans while still allowing these patients to continue to remain eligible for public funding. Measures were developed based on expert opinion from OH(CCO) tumor site groups, available evidence, and emerging pandemic guidance. A number of factors were considered, including: access to other treatment modalities (e.g., surgery, radiation, stem cell transplants) may be significantly delayed; hospital visits may need to be reduced due to patient risk or limited workforce capacity; less immunosuppressive therapies may be warranted due to patient risk; overall patient and budget impact; and the administrative burden of applying for drug coverage should be reduced for clinicians who may be facing an increased workload during the pandemic. Table S1 present a summary of the Ministry-approved interim funding measures. 

Following the first wave of the pandemic, we conducted an online survey on management strategies used by Ontario oncology clinicians to better understand the utility of the NDFP interim drug funding policies and the need for continuing these measures as the COVID-19 pandemic continues.

## 2. Experimental Section

### Methods

A self-administered English-language online questionnaire, consisting of 13 questions, was developed using Microsoft Forms (Office 365 E5). The survey addressed three topics: (1) What proportion of patients had their treatment plans modified? (2) What management strategies were used in the first wave of the pandemic? (3) Which management strategies did clinicians anticipate using in the next 3 months? Each interim funding measure was mapped to a specific survey question (see Table S1). 

The survey was pilot tested at the end of August 2020 by six hematologists and medical oncologists who were representative of the target population. Pilot testers were asked to assess the ease of use and clarity of questions, and questions were revised based on their feedback prior to broader distribution. 

An invitation to participate in the survey was emailed to Ontario clinicians via various distribution lists available at OH(CCO) that targeted medical oncologists, hematologists, pharmacists, and senior administrators of the regional cancer programs. Participants were also invited to share the survey with their peers. There were a total of 329 contacts on these distribution lists; however, there was some overlap between these lists and given the request to share the survey, the total number of unique invitees is not known. The survey was open between 3 and 25 September 2020.

Responses were collected anonymously. Personal information was not collected apart from voluntarily disclosed email addresses and sex. Survey responses were exported from Microsoft Forms into Excel, and responses were analyzed using descriptive statistics.

Comparisons of responses for treatments during the first wave versus the next three months were performed using the McNemar test, which accounts for the matched nature of the response by the participants for both questions. The results were considered exploratory with *p*-values < 0.05 considered statistically significant, and were not adjusted for multiple comparisons.

This work was conducted to evaluate and inform the utility of interim funding measures implemented by OH(CCO) to inform its administration of the NDFP, and as such, we did not seek ethics approval.

## 3. Results

### 3.1. Respondent Characteristics

A total of 105 responses were collected during the 22-day survey window and their characteristics are shown in Table 1. The three largest groups of respondents were medical oncologists (48%), pharmacists (21%), and hematologists (13%). Almost 60% of respondents have been in clinical practice for over 10 years. 

Table 2 displays the distribution of responses by practice setting, region in Ontario, and by tumor type. Responses were received from all regions of Ontario, encompassing urban and rural areas. Of the 72 systemic treatment facilities in the province, 33 different hospitals (46%) ranging from academic centres to community sites were represented. Among academic centres that can provide complex care, the centre-level response rate was very high (93%). There was representation from a cross-section of clinicians who manage a variety of solid tumors or hematological malignancies.

### 3.2. The Majority of Clinicians Modified Treatment Plans for Their Cancer Patients during the Covid-19 Pandemic

Between April and June 2020, 46% of respondents changed treatment plans for more than 25% of their cancer patients due to the pandemic (see Table 3). Five respondents (3/5 were urologists), from three different regions, modified treatment plans for more than 75% of their patients.

### 3.3. Clinicians Are Using Broad Range of Management Strategies and Will Continue to Do so in the Coming Months

**Figure 1:**
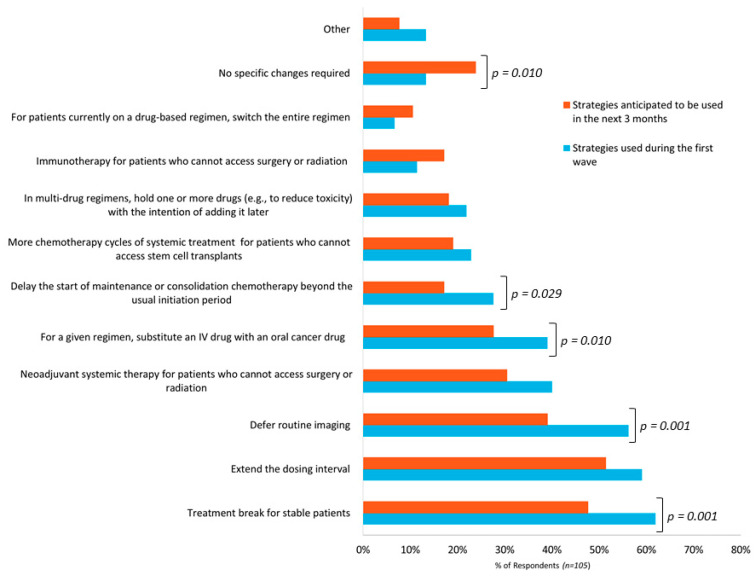
Frequency of strategies of used or anticipated to be used by respondents (*n* = 105) to manage cancer patients during the initial wave of the COVID-19 pandemic and in the upcoming three months.

Survey participants were asked to identify management strategies used during the initial wave of the pandemic and strategies they anticipated using in the next three months. Figure 1 depicts the responses from these two questions.

Regarding management strategies used during the initial wave, there were a total of 392 responses from the 105 participants. On average, respondents reported using at least three strategies. The most frequently reported strategies were giving a treatment break to stable patients (62%), extending the dosing interval (59%), and deferring routine imaging (56%). Of the 14 hematologists who responded to this survey, 11 (79%) gave more chemotherapy cycles to patients who could not access stem cell transplants. Thirteen percent of respondents reported not making any specific COVID-19-related treatment plan changes. Other strategies represented 13% of responses and included strategies such as deferring the use of intravenous zoledronic acid in patients with breast cancer (adjuvant or metastatic setting), prescribing refills on oral cancer medications that are typically not repeated without a physician visit, and the use of privately funded home injection programs to administer hormonal injections.

Regarding management strategies anticipated to be used in the upcoming three months, 325 responses were received from the 105 respondents. Respondents reported an average of three strategies that may be required. Similar to the initial wave, the most frequently anticipated strategies are giving a treatment break in stable patients (48%), extending the dosing interval (51%), and deferring routine imaging (39%). Compared to the respondent choices in the initial wave, fewer respondents anticipated the use of specific strategies and the difference was statically significant for several measures as illustrated in Figure 1. Furthermore, more respondents selected “no specific changes required” (13% versus 24% of respondents) and this difference was statistically significant. Other strategies reported included the use of home care for supportive therapies, more virtual follow-up appointments, and the use of radiation therapy instead of systemic therapy (e.g., for low grade non-Hodgkin lymphoma). Several respondents commented that anticipated strategies are not yet known due to uncertainty regarding the duration and impact of the pandemic.

### 3.4. Clinicians Require on-Going Support from the Cancer System for Optimal Patient Management during the Pandemic

As an optional question, participants were asked to identify which resources/guidelines they consulted to assist their decision-making during the pandemic. There were 101 respondents, who on average consulted two different resources. Most frequently consulted was the OH(CCO) pandemic guidance [13,14]. COVID-19 resources provided by BC Cancer and the American Society of Clinical Oncology were also frequently selected [16,17]. Other resources cited included the European Society of Medical Oncology, the American Society of Hematology, National Health Service, the Canadian Urological Association, and the National Comprehensive Cancer Network [18,19,20,21,22].

Participants were given an opportunity to provide additional comments, including identifying any additional funding measures that should be considered. While no additional COVID-19-specific potential funding changes were identified, respondents did identify pre-COVID-19 funding issues that may have become exacerbated during the pandemic.

Key themes identified (from 32 respondents):

- Flexibility in drug funding policy measures is required.
- Timely and efficient access to publicly funded drugs is important, especially for oral cancer drugs.
- Address current drug funding gaps to improve care during the COVID-19 pandemic.
- Addressing pre-pandemic funding issues remains important (e.g., drug wastage, care closer to home, physician shortages).

Beyond drug funding issues, several respondents cited challenges with the overall provision of virtual care for managing cancer patients.

## 4. Discussion

In Ontario, the “first wave” of the COVID-19 pandemic can be described as occurring between March and June 2020 where the province reported over 35,000 cases and 2841 related deaths [23]. During this period, the government enacted strict measures to control and manage the spread of the virus including ramping down scheduled surgeries (including cancer surgeries), temporary closures of schools and non-essential businesses, and restrictions on international travel [24,25]. Conducted after the initial wave of the pandemic, our survey collected responses that include robust representation across regions, practice settings, and clinical specialities. 

The uptake of each NDFP interim funding measure varies, suggesting that clinicians are taking an individualized approach to modifying patient treatment plans. In general, clinicians may consider multiple factors in their decision-making including: system-level constraints (e.g., hospital capacity), disease-specific factors (e.g., type of cancer), treatment intent, magnitude of treatment benefit, patient-specific factors (e.g., age, concomitant diseases, prognosis, risk of contracting COVID-19, risk of toxicity from a regimen), and patient preferences [9,10,18,19,26,27,28]. Since 89% of respondents in this survey consulted the OH(CCO) pandemic resources, we assume that their decision making was influenced by the priority classification proposed by OH(CCO).

In our survey, 51% of respondents reported using systemic therapy (i.e., immunotherapy or neoadjuvant therapy) when surgery or radiation was delayed. Systemic therapy was proposed as an option in the event that surgery and radiation were not readily available to patients during the pandemic [14,29]. Our findings suggest that this was a useful strategy. Ontario did experience limited access to surgery and radiation during the first wave, with 38% fewer cancer surgeries in April 2020 compared with April 2019 [11]. Breast cancer is a common cancer for which radiation therapy is needed. To reduce patient visits, Princess Margaret Cancer Centre (PMCC), the largest cancer centre in Canada, instituted mitigating measures for breast cancer patients who may require radiation therapy. Based on their oncologic risks, radiation therapy could be shortened, deferred, or omitted. During the first wave, PMCC reported a decrease of 39% in breast radiation therapy starts during March–April 2020, compared to the same period in 2019 [12,30]. During this time, the NDFP interim funding measures were available to support clinicians who wanted to prescribe adjuvant regimens in the neoadjuvant setting, or use systemic therapy where access to surgery or radiation was limited or not preferred.

Regarding the proportion of patients who had treatment plans modified, we found that 46% of respondents changed treatment plans for more than 25% of their patients during the initial wave (April–September 2020). This finding is slightly higher than was noted in a survey conducted by the Canadian Association of Medical Oncologists (CAMO) in May 2020, which showed that 21% of respondents reported changing chemotherapy plans for more than 20% of their patients [31]. There are a number of possible reasons for differences in these findings, including timing of the survey (our survey was conducted at a later stage of the pandemic), population surveyed (our survey included a broader group of clinicians other than medical oncologists), and geography (our survey was limited to Ontario). Of note, in a global survey of oncologists (*n* = 343) from 28 countries, 61% said that the COVID-19 outbreak would “definitely affect” treatment decisions, further highlighting the need to support clinicians with flexible public funding initiatives [27]. System-wide cancer drug funding policies must be flexible and responsive as the pandemic evolves. During the initial wave of COVID-19 in Ontario, the extent of lockdown measures varied by region [32]. Our survey was conducted a few months after the restart of all deferred and non-essential and elective services began to occur [33]. Broadly, respondents anticipated less use of specific COVID-19 management strategies in the coming months. Based on detailed comments provided from respondents, this may highlight clinician uncertainty regarding the extent of the restrictions that may be needed as the pandemic continues. A global survey of 351 cancer centres, conducted in April/May 2020, reported that 88.2% of centres reduced their usual care, and the most common reason was due to precautionary measures [10]. However, centres in Canada and other countries are also reporting fewer referrals from general practitioners to oncology providers for diagnosis or treatment [8,34,35]. This may mean Ontario could see a reduction in treatment volumes and the need for the interim funding measures in the coming months may be reduced.

In terms of specific treatment plan changes, the most frequently reported changes were giving a treatment break to patients who were stabilized on therapy, extending the dosing interval, and deferring routine imaging (Figure 1). These options reduce the overall consumption of healthcare resources, reduce the number of hospital visits for patients, and potentially reduce patient risk of contracting COVID-19. Usually, for these types of modifications, OH(CCO) requires prescribers to obtain patient-specific pre-approvals for NDFP funding. However, to reduce the administrative burden to clinicians, the requirement for prior authorization was waived.

During the pandemic, OH(CCO) permitted NDFP funding for re-starts, even if disease progression occurs while on a treatment break. In the United Kingdom, the National Institute for Health Care Excellence and the National Health Service issued similar guidance and funding policies for treatment breaks [19,36,37]. 

For many drugs and indications funded by the NDFP, imaging reports may be required to confirm patient eligibility at the start of therapy or for continued funding (e.g., to demonstrate a lack of disease progression while on therapy). For example, documentation indicating at least stable disease is usually required after every 12 cycles of Avastin (bevacizumab) for first line metastatic colorectal cancer. Considering the need to minimize hospital visits, OH(CCO) waived imaging requirements at the onset of the pandemic. This is certainly a driver for the high level of responses in our survey that indicated deferred routine imaging as a strategy.

In developing the interim funding measures, the need to extend the dosing intervals was highlighted by several tumour site groups. For example, in advanced melanoma, the policy is now permitting the use of pembrolizumab given less frequently but at a higher dose (i.e., 4 mg/kg IV every 6 weeks instead of 2 mg/kg IV every 3 weeks). BC Cancer has also suggested using immunotherapy protocols that are given at 4- or 6-week intervals, further highlighting the utility of this measure [26].

The COVID-19 pandemic has created unprecedented stress on cancer systems, and created new risks for cancer patients. Our survey findings indicate that OH(CCO) guidelines and funding policies have given clinicians options to adapt treatment plans considering the changing landscape of delivering cancer care during a pandemic. However, it is important to note that the outcomes of modifying treatment plans for cancer patients is not currently well established. When OH(CCO) first released the NDFP interim funding measures, clinicians were informed that interim cancer drug funding measures might not be appropriate for all patients. The onus was on prescribers to determine whether adopting a suggested interim funding measure for a given patient was clinically appropriate based on a thorough risk–benefit assessment. A recent patient survey conducted in the Netherlands found that more than 50% of patients were concerned about the consequences of delays and discontinuation of treatments [38]. These patient concerns are validated in a recent systematic review showing that “… a four week delay of cancer treatment is associated with increased mortality across surgical, systemic treatment, and radiotherapy indications for seven cancers” [39]. Collectively, these findings highlight the need to closely monitor the COVID-19 pandemic, including the impact of treatment adaptations, to assess the overall impact on cancer outcomes.

Our study had several limitations. We surveyed clinicians on pandemic management strategies to infer the presumed utility of the changes to the NDFP funding policies. A response rate could not be calculated because of overlapping membership between our distribution lists. While we received responses from all 14 regional cancer programs, the limited sample size means that certain regions and oncology specialties may be over- or under-represented. The representation by provider type was not evenly distributed across the regions so we were not able to make meaningful comparisons. For example, physicians, pharmacists, and nurses may have different perspectives depending on the information that is directly available to them. Additionally, respondents may differ in their level of clinical or administrative duties, so some respondents (e.g., pharmacists) may be describing centre-level practice while most respondents would be describing their individual practices.

A detailed quantitative analysis using administrative health data to examine changes in practice, associated morbidity and mortality, and system impact would give a more accurate determination of the utilization and effects of the interim funding measures. While the results of our survey illustrate how Ontario clinicians are adapting treatment plans primarily for cancer patients on systemic therapies, we did not examine how treatment strategies differ for existing versus newly diagnosed patients.

## 5. Conclusions

Our report examines the utility of several interim cancer drug funding measures designed to provide Ontario patients with access to therapies that may be needed as clinicians adapt treatment plans during the COVID-19 pandemic. Overall we found that the majority of clinicians are modifying treatment plans, and are utilizing the interim funding measures. These findings may be broadly useful to policymakers and providers in other jurisdictions who are involved in cancer care.

As the pandemic evolves policymakers should consider the on-going impact of COVID-19 on cancer drug funding policies, and their subsequent effects on cancer providers and patients. Regular reassessment of clinical practices and funding policies, in this era of COVID-19, may be required to support the continued delivery of high-quality care.

## Figures and Tables

**Table 1:** Respondent demographics (*n* = 105).

Parameter	Result
Sex, *n* (%)	
Female	48 (45.7%)
Male	52 (49.5%)
Not specified	5 (4.8%)
Provider Type, *n* (%)	
Physicians	79 (75.2%)
Medical Oncologist	50 (47.6%)
Hematologist	14 (13.3%)
Urologist	10 (9.5%)
Gynecology Oncologist	2 (1.9%)
Surgical Oncologist	1 (1.0%)
Radiation Oncologist	1 (1.0%)
General Practitioner in Oncology	1 (1.0%)
Pharmacist	22 (21.0%)
Nurse	3 (2.9%)
Other	1 (1.0%)
Years in Practice, *n* (%)	
<5 years	26 (24.8%)
5–10 years	19 (18.1%)
10–15 years	19 (18.1%)
>15 years	41 (39.0%)

**Table 2:** Distribution of responses by practice setting, region, and tumor type (*n* = 105).

Parameter	Result
Practice Setting, *n* (%)	
Level 1—Regional Cancer Centre (academic) ^1^	43 (41.0%)
Level 2—Regional Cancer Centre (academic)	25 (23.8%)
Level 3—Affiliate centre (community)	29 (27.6%)
Level 4—Satellite site ^2^ (community)	8 (7.6%)
Regions of Ontario, *n* (%)	
1. Erie St. Clair	2 (1.9%)
2. South West	3 (2.9%)
3. Waterloo Wellington	14 (13.3%)
4. Hamilton Niagara Haldimand Brant	16 (15.2%)
5. Central West	4 (3.8%)
6. Mississauga Halton	3 (2.9%)
7. Toronto Central	15 (14.3%)
8. Central	5 (4.8%)
9. Central East	12 (11.4%)
10. South East	9 (8.6%)
11. Champlain	11 (10.5%)
12. North Simcoe Muskoka	4 (3.8%)
13. North East	5 (4.8%)
14. North West	2 (1.9%)
Tumor Types Treated, count (%) ^3^	
All Solid Tumors	21 (8.4%)
All hematology malignancies	29 (11.6%)
Breast	28 (11.2%)
CNS	4 (1.6%)
Genitourinary	32 (12.8%)
Gastrointestinal	30 (12.0%)
Gynecologic	17 (6.8%)
Head and Neck	7 (2.8%)
Lung	30 (12.0%)
Leukemia	9 (3.6%)
Lymphoma	11 (4.4%)
Myeloma	8 (3.2%)
Melanoma	20 (8.0%)
Other	4 (3.8%)

^1^ Level 1 centres have a robust experimental Investigational New Drug Program [15]. ^2^ Level 4 centres execute systemic treatment plans under the direction of an oncologist from a level 1–3 centre [15]. ^3^ Frequency based on 250 responses by 105 respondents. Some may treat more than one tumor type.

**Table 3:** Proportion of patients requiring treatment plan modifications between April and June 2020 due to the COVID-19 pandemic.

Proportion of Patients Requiring Treatment Plan Changes	No. of Respondents (%)
<25%	57 (54.3%)
25–50%	35 (33.3%)
50–75%	8 (7.6%)
>75%	5 (4.8%)

## Data Availability

The data presented in this study are available on request from the corresponding author. The data are not publicly available due to program and privacy requirements.

## Supplementary Materials

The following are available online https://www.mdpi.com/1718-7729/28/2/103/s1. Table S1: Interim funding measures for hospital administered cancer drugs funded by the New Drug Funding Program (NDFP). Implemented 30 March 2020 and last updated 31 July 2020. For the purposes of surveying clinicians, each funding measure was mapped to a category used for the survey questions.
