# Supplementary material for: Clinician Perspectives of COVID-19-Related Cancer Drug Funding Measures in Ontario
Source: Curr Oncol. 2021 Feb 26;28(2):1056–66. doi: 10.3390/curroncol28020103 (PMC8025744; doi:10.3390/curroncol28020103)
Supplement: Supplementary file 1 [file curroncol-28-00103-s001.pdf]

Supplementary Material

Table 1. Interim funding measures for hospital administered cancer drugs funded by the New Drug Funding Program (NDFP). Implemented March 30, 2020 and last updated July 31, 2020. For the purposes of surveying clinicians, each funding measure was mapped to a category used for the survey questions.

Interim Funding Measure	Explanation	Examples	Category for Survey Questions
1.Extended Treatment Breaks	For patients that take a treatment break during the pandemic, NDFP will fund re-starts, even if disease progression occurs while on the break.	Nivolumab – previously treated metastatic renal cell carcinoma: consider treatment break for stable patients<br><br>Nivolumab or pembrolizumab - advanced non-small cell lung cancer: consider treatment break for stable patients at the 6 month plus mark	Gave a treatment break to patients who are stable
2. Flexible start dates for sequencing treatments	For circumstances where a systemic treatment must be started within a certain time period after another intervention.<br><br>A delay in the start date of the systemic treatment will not affect a patient's eligibility for public drug funding coverage.	Rituximab - maintenance treatment for lymphoma: usually start within 6 months of completing induction therapy.<br><br>Brentuximab - consolidation post- autologous stem cell transplant (ASCT) for Hodgkin lymphoma: usually start 4-6 weeks after ASCT or upon recovery from ASCT.	Delayed the start of maintenance or consolidation chemotherapy beyond the usual initiation period *(e.g., rituximab treatment for lymphoma)*
3. Extended dosing intervals	Extended dosing intervals will be approved for specific regimens.	Pembrolizumab – previously treated locally advanced or metastatic urothelial carcinoma; previously untreated locally advanced metastatic non-small cell lung cancer;	Extended the dosing intervals of drugs, or gave drugs less frequently *(e.g., give a drug every 6 weeks instead of every 3 weeks)*

		advanced melanoma: 4mg/kg IV (up to a maximum of 400mg) every 6 weeks as an alternative option. *{usual funded dose: 2mg/kg IV every 3 weeks up to a maximum of 200mg/dose}*<br><br>Durvalumab - locally advanced unresectable Stage III non-small cell lung cancer following concurrent chemoradiation: 20mg/kg IV (up to a maximum of 1500mg) every 4 weeks as an alternative option. *{usual funded dose: 10mg/kg IV every 2 weeks up to a maximum of 12 months}*	
4.Reduced documentation for imaging requirements	No requirement to provide CT scan results for continued funding.	Avastin (bevacizumab) for first-line metastatic colorectal, small bowel, or appendiceal cancer: documentation indicating at least stable disease is required every 12 cycles. This requirement is waived during the COVID-19 pandemic.<br><br>Brentuximab vedotin for relapsed/refractory Hodgkin lymphoma: treatments beyond 16 cycles require documentation showing continued evidence of benefit (i.e., a clinic note and CT scan confirming that there is no evidence of disease progression). This requirement is waived during the COVID-19 pandemic.	Deferred routine imaging
5.Extend adjuvant funding to neo-adjuvant	All treatments typically funded in the adjuvant setting will also be funded in the neo-adjuvant setting.	Nivolumab - adjuvant treatment for completely resected Stage III or IV melanoma - both adjuvant and neo-adjuvant treatments will be funded by NDFP.	Used neoadjuvant systemic therapy for patients who cannot access surgery or radiation.

	Paclitaxel in combination with platinum - first line – advanced ovarian, fallopian tube, or primary peritoneal carcinoma: both adjuvant and neo-adjuvant treatments will be funded by NDFP.	

**7. Treatment algorithm modifications due to capacity constraints**	Funding and treatment algorithms can be modified without affecting funding.	Bortezomib – previously untreated, multiple myeloma, pre- autologous stem cell transplant (ASCT): the NDFP will fund up to 9 cycles of bortezomib (as part of CyBor-D) pre-transplant in patients who have their autologous stem cell transplant (ASCT) delayed.<br><br>Nivolumab – Recurrent or metastatic squamous cell carcinoma of the head and neck, which is platinum resistant or refractory: if there is no access to surgery or radiation and treatment intent becomes palliative, NDFP will fund.<br><br>Metastatic castration resistant prostate cancer who are docetaxel naïve or where continuation of docetaxel is deemed to be inappropriate during the pandemic: consider a second androgen receptor-axis-targeted agent [ARAT] (e.g., enzalutamide) if good response to a prior ARAT (e.g.., abiraterone). Upon disease progression (and resolution of the pandemic), NDFP will considering funding for cabazitaxel (or docetaxel, where applicable).	Gave more chemotherapy cycles of systemic treatment for patients that cannot access stem cell transplants<br><br>Used immunotherapy for patients who cannot access surgery or radiation *(e.g., nivolumab for head & neck cancer)*<br><br>For a given regimen, substituted an IV drug with an oral cancer drug *(e.g., when treating prostate cancer or myeloma)*

**8. Allowing subsequent funding for drug(s) that are temporarily held within a regimen**	One or more drugs in a regimen may be held and added later, even if there is disease progression on the modified regimen.	Nivolumab plus ipilimumab - advanced melanoma:<br><br>For patients who would normally be treated with combination ipilimumab plus nivolumab, if clinician discretion dictates that the use of ipilimumab needs to be deferred, NDFP will fund the addition of ipilimumab at a later date.	Held one or more drugs (e.g., to reduce toxicity) with the intention of adding it later, in patients currently on multi-drug regimens *(e.g., held ipilimumab for melanoma patients on nivolumab/ipilimumab*)
**9. Funding switches to alternative treatment options**	Clinicians may switch to an alternative treatment option	Nivolumab – adjuvant treatment for completely resected Stage III or IV melanoma:<br><br>Since dabrafenib-trametinib can cause fevers that may be mistaken for COVID-19, consider switching adjuvant dabrafenib-trametinib to adjuvant immunotherapy (e.g., nivolumab)	For patients currently on a drug-based regimen, switched the entire regimen *(e.g., switch melanoma patients from adjuvant dabrafenib/trametinib to immunotherapy)*
